# Parameter-Free Statistical Generator-Based Class Incremental Learning for Multi-User Physical Layer Authentication in the Industrial Internet of Things

**DOI:** 10.3390/s25195952

**Published:** 2025-09-24

**Authors:** Wanbing Zhao, Yanru Guo, Yuchen Huang, Yanru Chen, Liangyin Chen

**Affiliations:** 1 College of Computer Science, Sichuan University, Chengdu 610065, China; wanbingzhao@stu.scu.edu.cn (W.Z.); guoyanru@stu.scu.edu.cn (Y.G.); yuchen_huang@stu.scu.edu.cn (Y.H.); chenyanru@scu.edu.cn (Y.C.); 2 Institude for Industrial Internet Research, Sichuan University, Chengdu 610065, China

**Keywords:** industrial internet of things, multi-user physical layer authentication, class incremental learning

## Abstract

Deep learning (DL)-based multi-user physical layer authentication (PLA) in the Industrial Internet of Things (IIoT) requires frequent updates as new users join. Class incremental learning (CIL) addresses this challenge, but existing generative replay approaches depend on heavy parameterized models, causing high computational overhead and limiting deployment in resource-constrained environments. To address these challenges, we propose a parameter-free statistical generator-based CIL framework, PSG-CIL, for DL-based multi-user PLA in the IIoT. The parameter-free statistical generator (PSG) produces Gaussian sampling on user-specific means and variances to generate pseudo-data without training extra models, greatly reducing computational overhead. A confidence-based pseudo-data selection ensures pseudo-data reliability, while a dynamic adjustment mechanism for the loss weight balances the retention of old users’ knowledge and the adaptation to new users. Experiments on real industrial datasets show that PSG-CIL consistently achieves superior accuracy while maintaining a lightweight scale; for example, in the AAP outer loop scenario, PSG-CIL reaches 70.68%, outperforming retraining from scratch (58.57%) and other CIL methods.

## 1. Introduction

With the rapid proliferation of Industrial Internet of Things (IIoT) devices and the openness of electromagnetic wave propagation, efficient and secure wireless authentication has become crucial for IIoT scenarios [1]. Compared to traditional upper-layer authentication and threshold-based Physical Layer Authentication (PLA) [2,3], Deep Learning (DL)-based multi-user PLA leverages DL models to automatically extract features from channel characteristics, such as the Channel Impulse Response (CIR) and Channel State Information (CSI) of different users (i.e., devices), demonstrating promising potential for more robust and scalable authentication [4,5,6,7,8]. However, as new users are continuously added in realistic IIoT scenarios, the DL model must frequently update to maintain authentication performance. A straightforward approach is retraining the model from scratch using both old and new users’ data. However, this approach is impractical under the stringent real-time latency constraints and limited on-device storage typical of IIoT systems, because retraining from scratch incurs substantial model-update latency and requires the retention of historical user data [9].

Compared to retraining from scratch, class incremental learning (CIL) offers a more feasible paradigm. Since the currently trained model already retains authentication knowledge of old users, CIL expands the fully connected layer of the model and incrementally fine-tunes it using new users’ data [10], thereby enabling rapid adaptation to new user authentication without storing old users’ data. To mitigate catastrophic forgetting in class incremental learning, a phenomenon of significantly forgetting the knowledge of old users after adapting to new users, existing CIL methods primarily employ two strategies: regularization and generative replay. Regularization-based CIL implicitly introduces additional loss terms, such as knowledge distillation, to prevent deviations of model parameters from previous knowledge [11,12,13]. In contrast, generative replay-based CIL explicitly employs additional parametric generative models like Generative Adversarial Networks (GANs) to produce pseudo-data for old users [14,15,16], thereby better preserving user decision boundaries and achieving superior performance in mitigating catastrophic forgetting [10]. Moreover, these generative models can augment new user data [17,18], alleviating overfitting caused by limited data availability in IIoT scenarios [5,6]. However, the training and inference of additional parametric generative models require substantial computational costs, limiting their deployment in resource-constrained IIoT scenarios [19]. Despite these advances, several key challenges remain in applying CIL to multi-user PLA in IIoT scenarios. New users frequently join the system, which requires efficient model updates without relying on old user data. At the same time, incremental methods often encounter catastrophic forgetting, where previously acquired knowledge is overwritten when adapting to new users. Moreover, generative replay methods depend on parameterized models, resulting in substantial computational overhead that is impractical for resource-constrained IIoT devices. These issues collectively highlight the need for a lightweight yet effective incremental learning framework.

To address these challenges, we propose a parameter-free statistical generator-based CIL (PSG-CIL) framework, which can be seamlessly integrated into the existing DL-based multi-user PLA model. Specifically, the parameter-free statistical generator (PSG) produces pseudo-data for old users by sampling from a Gaussian distribution constructed using two statistical features, namely the mean and variance of user data, thus avoiding additional parameter training and greatly reducing computational overhead. Furthermore, we introduce a data selection mechanism to ensure that the pseudo-data generated by PSG maintains high reliability. PSG is also able to augment new users’ data in a similar manner, mitigating the risk of overfitting caused by limited data availability. To better balance the retention of old users’ knowledge and the adaptation to new users, we further adopt a weighted loss function that combines knowledge distillation and cross-entropy, along with a dynamic adjustment mechanism for the loss weight. Extensive experiments on real industrial scenario datasets [20] demonstrate that PSG-CIL achieves the best authentication accuracy compared to retraining from scratch and other CIL methods, demonstrating its efficiency and adaptability in IIoT scenarios. Our contributions are summarized as follows:

- We propose PSG-CIL, a parameter-free CIL framework that can be seamlessly integrated into existing DL-based multi-user PLA models. Compared to existing generative replay-based CIL methods, PSG-CIL generates reliable pseudo-data for old users without any additional parameter training, thereby maintaining authentication performance while significantly reducing computational overhead.
- PSG is capable of augmenting new users’ data simultaneously, mitigating overfitting caused by limited data availability and further improving authentication accuracy.
- We further introduce a dynamic loss weight adjustment mechanism, enabling a more flexible balance between retaining old users’ knowledge and adapting to new users.
- Extensive experiments on real industrial scenario datasets demonstrate the high efficiency and adaptability of PSG-CIL on real IIoT scenarios. For example, in the outer loop route of the Automotive Assembly Plant (AAP) scenario [20] with a Convolutional Neural Network (CNN) as the unified training DL model, when the total number of users reaches seven, retraining from scratch achieves accuracies around 58.57%, while our PSG-CIL framework achieves 70.68%.

The remainder of this article is organized as follows. A literature review of DL-based multi-user PLA and CIL is presented in Section 2. In Section 3, the system model and problem formulation are provided. Section 4 provides the PSG-CIL framework. Performance evaluation is presented in Section 5 with conclusions in Section 6.

## 2. Related Work

### 2.1. DL-Based Multi-User PLA

Recent advances in intelligent physical layer authentication have applied both deep learning (DL) and artificial intelligence (AI) techniques to enhance multi-user PLA. DL-based multi-user PLA formulates authentication as a multi-class classification task and trains deep models to capture user-specific physical layer channel characteristics [8,21,22,23]. Compared with traditional PLA methods, DL-based methods offer superior fitting and classification capabilities for multi-user PLA, which have gradually attracted increasing research attention [3,24,25]. Liao et al. [5] proposed multi-user PLA methods based on a Deep Neural Network (DNN) and CNN, respectively, while using data augmentation techniques to alleviate the problem of not having enough data to train the network model. Xia et al. [26] proposed a new multi-attribute authentication scheme. It uses non-parametric clustering and heuristic algorithms to account for the correlations among multiple attributes at the physical layer. Meng et al. [6] introduced Gaussian noise into the smoothed perturbed space, and proposed the Lightweight Perturbed Convolutional Neural Network (LPCNN) to improve the authentication performance while reducing the requirement for the amount of training data. Jing et al. [7] further used the more complex Residual Network (ResNet) model and parameter-based transfer learning approach to improve the accuracy of multi-user authentication in mobile scenarios. Wu et al. [27] proposed a CANN model that integrates an attribute attention module into a CNN to extract more unique features of users from multiple physical layer attributes.

In parallel, AI-assisted PLS has also been explored in broader wireless security contexts. For example, asynchronous deep reinforcement learning has been applied to optimize NOMA-assisted secure offloading in vehicular edge computing networks [28], while deep recurrent reinforcement learning has been utilized for energy-efficient cooperative secure communications in mmWave vehicular networks [29]. These studies demonstrate the potential of AI-assisted approaches for enhancing PLS under vehicular scenarios. However, they mainly target secure offloading or cooperative communication rather than user authentication, and are oriented toward vehicular networks. In contrast, our work focuses on incremental multi-user PLA in IIoT, where frequent user arrivals and strict resource constraints make lightweight incremental learning particularly crucial.

However, most of these studies focus on improving model architectures or feature extraction while neglecting the dynamic nature of IIoT scenarios where new users continuously join.

### 2.2. Class Incremental Learning

CIL extends the current trained model to accommodate new classes while retaining previously learned knowledge without storing old class data. To address catastrophic forgetting, two primary approaches have been explored: regularization-based methods and generative replay-based methods. Regularization-based methods implicitly constrain model updates to prevent drastic deviations from learned representations, ensuring old knowledge is preserved while integrating new information. Li et al. [11] introduced knowledge distillation to maintain consistency between old and new class representations. Hu et al. [13] incorporated causal inference to enhance incremental adaptation and improve performance. Liu et al. [12] developed an adaptive feature extraction mechanism, allowing the model to dynamically balance stability and plasticity as new classes are introduced. Generative replay-based methods introduce additional parametric generative models, such as GAN [14,16], to explicitly produce pseudo-data for old classes, thereby better preserving user decision boundaries compared to regularization-based methods and achieving superior performance in mitigating catastrophic forgetting. Petit et al. [18] further leveraged generative models to augment new class data, enhancing feature diversity and preventing the model from excessively fitting new users’ data. Such methods have also been effectively applied in some real-world applications, such as human activity recognition [30] and audio–video processing [31]. However, the computational costs of training additional complicated generative models could make them less suitable for resource-constrained IIoT scenarios [19]. Nevertheless, regularization-based methods often fail to sufficiently preserve old knowledge, while generative replay-based methods impose heavy training costs, limiting their scalability and deployment in IIoT environments.

## 3. System Model and Problem Formulation

### 3.1. System Model

**Figure 1:**
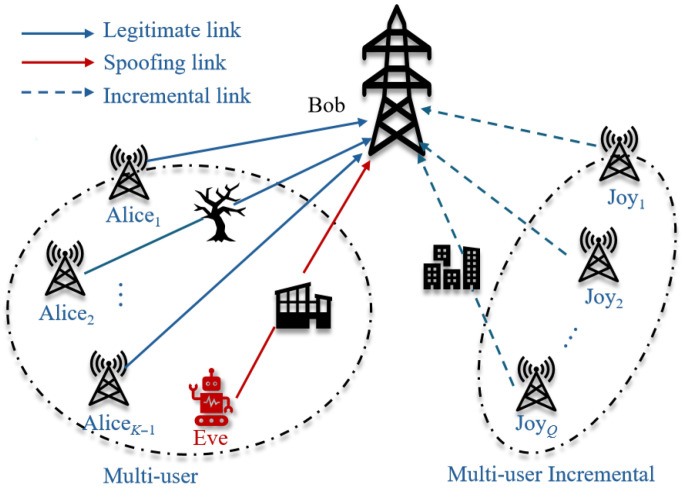
Fixed multi-user PLA and incremental multi-user PLA in the IIoT scenarios.

As illustrated in Figure 1, multi-user PLA leverages the distinctive physical channel characteristics of users to accurately identify user identities, ensure the access rights of legitimate users, and prevent attacks or signal interception by unauthorized users. In this work, the CIR is adopted as the primary physical layer feature for authentication [6,27,32], as it is inherently user-specific due to the combined effects of multipath propagation, path loss, and shadow fading at different user locations. Formally, in a *K*-users PLA system in IIoT, illegitimate users (e.g., attackers or eavesdroppers) are represented as Eve, while legitimate users are denoted as ${\mathrm{Alice}}_{1},{\mathrm{Alice}}_{2},\ldots ,{\mathrm{Alice}}_{K-1}$. In this work, following [6,32], users are identified through upper-layer authentication mechanisms and the system’s initial enrollment phase. Each user transmits pilot signals to the base station, and the corresponding CIR measurements are stored as reference data for subsequent authentication. Given a CIR vector *h* at a specific time instant *t*, the set of *N* different time slots for the *k*-th user is represented as $H_{k}=\left\{h_{k}^{1},h_{k}^{2},\ldots ,h_{k}^{N}\right\}$. Consequently, the dataset consisting of CIR data from *K* users can be denoted as $H=\{H_{1},H_{2},\ldots ,H_{K}\}$ with the corresponding labels Y={0,1,…,K−1}. By comparing CIR vectors across users, the system can effectively differentiate user identities, enabling robust authentication and enhancing overall system security.

### 3.2. DL-Based Multi-User PLA

In DL-based multi-user PLA methods, the process is divided into three sequential stages: data processing, model training, and authentication.

#### 3.2.1. Data Processing

For the *k*-th user at time slot *t*, the original CIR vector is denoted as a complex-valued vector $h_{k}^{t}={\left[h_{k,1}^{t},h_{k,2}^{t},\ldots ,h_{k,L}^{t}\right]}^{T}$, where $h_{k,\ell }^{t}=a_{k,\ell }^{t}+j\;b_{k,\ell }^{t}$ is the ℓ-th tap of the CIR (ℓ∈{1,…,L}), and $a_{k,\ell }^{t}$ and $b_{k,\ell }^{t}$ are its real and imaginary components, respectively. To accommodate the real-valued input requirement of DL models, each complex CIR vector is converted into real-valued representation by concatenating the real and imaginary parts followed by [6]:

(1) $$h_{k}^{t}={\left[a_{k,1}^{t},a_{k,2}^{t},\ldots ,a_{k,L}^{t},\;b_{k,1}^{t},b_{k,2}^{t},\ldots ,b_{k,L}^{t}\right]}^{T}.$$

This approach preserves both amplitude and phase information, which is critical for physical layer authentication.

#### 3.2.2. Model Training

In DL-based multi-user PLA, the model architecture typically comprises a feature extractor and a classifier. The feature extractor learns discriminative representations from the CIR signals, often employing DNN, CNN, or their enhanced variants to capture spatial and temporal dependencies across CIR taps. To fit the input format of CNNs, the processed CIR data is organized into a three-dimensional tensor $h\in R^{C\times L\times 2}$, where *C* denotes the number of replicated or attribute channels, *L* is the number of CIR taps, and the last dimension stores the real and imaginary components. The extracted representations are then passed through a Fully Connected (FC) layer, mapping them to the corresponding user classes. A softmax activation function is subsequently applied to produce a probability distribution over all *K* users, enabling multi-class classification.

The model is trained using cross-entropy loss to measure the discrepancy between the predicted and true labels:

(2) $$L_{\mathrm{CE}}=-\sum_{k=1}^{K}\sum_{h\in H_{k}}\sum_{i=1}^{K}\delta_{y,i}\log f_{i}\left(h\right),$$

where $f_{i}\left(h\right)$ is the predicted probability of class *i*, *y* is the true label of sample *h*, and $\delta_{y,i}$ is the Kronecker delta, equal to 1 if y=i and 0 otherwise. By minimizing $L_{\mathrm{CE}}$, the model parameters are updated via backpropagation and gradient-based optimization, thereby reducing classification errors and improving authentication accuracy.

#### 3.2.3. Online Authentication

Once the training phase is completed, the optimized model is deployed for real-time authentication. The newly received CIR samples are first preprocessed and then fed into the trained model to identify the corresponding user. Formally, the predicted class label $\hat{y}$ for a received CIR sample *h* is determined by

(3) $$\hat{y}=\underset{k\in \{1,2,\ldots ,K\}}{\arg\max}f_{k}\left(h\right),$$

where $f_{k}\left(h\right)$ represents the probability assigned by the model to user class *k*. In this way, the system verifies the identity of legitimate users while rejecting unauthorized ones.

### 3.3. Problem Formulation

In IIoT scenarios, the number of authenticated users is dynamic, as new users may continuously join the system over time. Formally, let *Q* new users, denoted as ${\mathrm{Joy}}_{1},{\mathrm{Joy}}_{2},\ldots ,{\mathrm{Joy}}_{Q}$, be added to the existing authentication system. The CIR dataset of these new users is represented as $H_{\mathrm{new}}=\left\{H_{1},H_{2},\ldots ,H_{Q}\right\}$ with the corresponding labels $Y_{\mathrm{new}}=\{K,K+1\;\ldots ,K+Q$−1}, where *K* denotes the number of previously enrolled users. The labels are assigned incrementally to accommodate the expanding user base.

To incorporate the authentication of new users, CIL expands the existing DL-based model $f_{\mathrm{old}}$ and updates it by fine-tuning on $H_{\mathrm{new}}$. This enables the model to efficiently adapt to both old and new users without requiring access to previously collected user data, thereby avoiding retraining from scratch. However, existing CIL methods often struggle to balance authentication accuracy and computational efficiency in IIoT scenarios, due to the introduction of additional trainable parameters and the tendency to overfit to new user data. To overcome these challenges, this work focuses on developing a novel CIL method that achieves high authentication accuracy while avoiding the introduction of extra trainable parameters.

## 4. The Proposed PSG-CIL Framework

**Figure 2:**
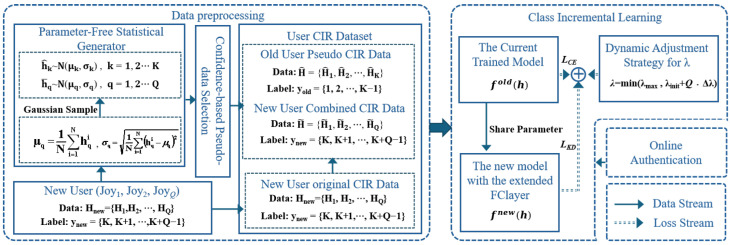
Overview of the PSG-CIL framework. In the Data Preprocessing stage, high-confidence pseudo-samples are generated through the parameter-free statistical generator (PSG) combined with a confidence-based pseudo-data selection mechanism. In the CIL stage, a dynamic adjustment strategy ensures a more flexible balance between retaining old users’ knowledge and adapting to new users during model updates.

This section presents the PSG-CIL framework for DL-based multi-user PLA in IIoT scenarios. The framework is composed of three core components. First, a parameter-free statistical generator (PSG) is introduced to generate pseudo-data for previously enrolled users and to augment the data of new users, thereby alleviating data scarcity. Second, a Confidence-based Pseudo-data Selection mechanism is designed to ensure the reliability of pseudo-data corresponding to old users. Third, a Class Incremental Learning (CIL) procedure is employed to seamlessly integrate new users while preserving knowledge of old users, thus avoiding the need for retraining from scratch. An overview of the proposed framework is illustrated in Figure 2, and the details of each component are elaborated on in the following subsections.

### 4.1. Parameter-Free Statistical Generator

To efficiently generate pseudo-data for old users while minimizing computational overhead in resource-constrained IIoT scenarios, we introduce the parameter-free statistical generator (PSG). Unlike parameterized generative models such as GANs or Variational Autoencoder (VAE), which require extensive training and high computational resources, PSG exploits simple statistical descriptors, namely the mean and variance, to generate pseudo-data through Gaussian sampling. Owing to its parameter-free nature, PSG can also be directly applied to augment the data of new users, thereby mitigating overfitting and improving authentication accuracy.

Specifically, let $H_{q}=\left\{h_{q}^{1},h_{q}^{2},\ldots ,h_{q}^{N}\right\}$ denote the CIR data collected for a new user *q*. The mean $\mu_{q}$ and variance $\sigma_{q}$ are computed as:

(4) $$\mu_{q}=\frac{1}{N}\sum_{i=1}^{N}h_{q}^{i},\;\sigma_{q}=\sqrt{\frac{1}{N}\sum_{i=1}^{N}{\left(h_{q}^{i}-\mu_{q}\right)}^{2}}.$$

These two statistics serve as the memory representation of user *q*. Similarly, for each old user *k*, PSG stores its mean $\mu_{k}$ and variance $\sigma_{k}$. Based on these statistics, pseudo-data for old users and augmented data for new users are generated via Gaussian sampling:

(5) $$\begin{array}{cc} {\hat{h}}_{k} & ∼N(\mu_{k},\sigma_{k}),\;k=1,2,\ldots ,K,\\ {\tilde{h}}_{q} & ∼N(\mu_{q},\sigma_{q}),\;q=1,2,\ldots ,Q, \end{array}$$

where ${\hat{h}}_{k}$ denotes the pseudo-data of the *k*-th old user, and ${\tilde{h}}_{q}$ denotes the augmented data for the *q*-th new user. To balance the number of data per user, *M* pseudo-data are generated for each old users, while for the new users, we also construct a final set of size *M* by combining the original *N* data with *M* − *N* augmented data, expressed as:

(6) $$\begin{array}{cc} {\hat{H}}_{k} & =\{{\hat{h}}_{k}^{1},\ldots ,{\hat{h}}_{k}^{N},{\hat{h}}_{k}^{N+1},\ldots ,{\hat{h}}_{k}^{M}\},\\ {\tilde{H}}_{q} & =\{h_{q}^{1},\ldots ,h_{q}^{N},{\tilde{h}}_{q}^{N+1},\ldots ,{\tilde{h}}_{q}^{M}\}. \end{array}$$

Since Gaussian sampling requires only minimal computational time and resources, PSG is particularly suitable for deployment in resource-constrained IIoT scenarios.

### 4.2. Confidence-Based Pseudo-Data Selection

Although PSG provides a fast and parameter-free way to generate pseudo-data, the sampled instances may not always faithfully reflect the characteristics of old users. To address this issue, we introduce a confidence-based selection mechanism that ensures only highly reliable pseudo-data are retained. Specifically, the currently trained model $f^{old}$ is employed to evaluate whether a pseudo-data point ${\hat{h}}_{k}^{m}$ can be accepted for the *k*-th old user. A pseudo-data sample is preserved only if its likelihood at the *k*-th output position of $f^{old}$ exceeds a predefined confidence threshold τ:

(7) $$f_{k}^{old}\left({\hat{h}}_{k}^{m}\right)>\tau ,\;m=1,2,\ldots ,M,$$

where $f_{k}^{old}\left({\hat{h}}_{k}^{m}\right)$ denotes the probability assigned by $f^{old}$ that ${\hat{h}}_{k}^{m}$ belongs to the *k*-th old user class, and τ is the confidence threshold.

### 4.3. Multi-User Incremental Learning

Once the data preprocessing is completed, all the data are fed into the CIL procedure to efficiently adapt the current trained model $f^{old}$ to new users while preserving previously learned knowledge without requiring retraining from scratch. First, we expand the FC layer of $f^{old}$ to accommodate *Q* new users classes. Specifically, the original FC layer, which consists of a weight matrix $W_{old}$∈$R^{K\times D}$ and a bias vector $b_{old}$∈$R^{K}$, is extended by adding *Q* additional rows as $W_{new}$∈$R^{(K+Q)\times D}$ and $b_{new}$∈$R^{K+Q}$. The rest of the model remains unchanged, and the new model with the extended FC layer is denoted as $f^{new}$. Consequently, for a CIR data point *h*, the outputs of the old model and new model can be denoted as:

(8) $$\begin{array}{c} \begin{array}{c} f^{old}\left(h\right)=\left[f_{1}^{old}\left(h\right),\ldots ,f_{K}^{old}\left(h\right)\right],\\ f^{new}\left(h\right)=\left[f_{1}^{new}\left(h\right),\ldots ,f_{K}^{new}\left(h\right),\ldots ,f_{K+Q}^{new}\left(h\right)\right]. \end{array} \end{array}$$

By retaining the feature extractor from $f^{old}$, the new model $f^{new}$ can quickly adapt to authenticate new users without retraining from scratch. To further stabilize the optimization, we fine-tune $f^{new}$ with a lower learning rate on the feature extractor to preserve previously learned knowledge and a higher learning rate on the expanded FC layer to quickly adjust for the new users classes. To mitigate catastrophic forgetting when updating the model, we also leverage a Knowledge Distillation (KD) loss $L_{\mathrm{KD}}$ [11], which ensures that the predictions of $f^{new}$ remain aligned with those of $f^{old}$. The KD loss is formulated as follows:

(9) $$\begin{array}{c} \begin{array}{c} L_{\mathrm{KD}}=\sum_{k=1}^{K}\sum_{h\in {\hat{H}}_{k}}\sum_{i=1}^{K}-\frac{f_{i}^{old}\left(h\right)}{T}\log\frac{f_{i}^{new}\left(h\right)}{T}\\ +\sum_{q=1}^{Q}\sum_{h\in {\tilde{H}}_{q}}\sum_{i=1}^{K}-\frac{f_{i}^{old}\left(h\right)}{T}\log\frac{f_{i}^{new}\left(h\right)}{T}, \end{array} \end{array}$$

where *T* is a temperature scaling parameter to smooth the output distributions. Meanwhile, the cross-entropy loss $L_{\mathrm{CE}}$ is employed to supervise the classification of both new and old users data:

(10) $$\begin{array}{c} \begin{array}{c} L_{\mathrm{CE}}=\sum_{k=1}^{K}\sum_{h\in {\hat{H}}_{k}}\sum_{i=1}^{K+Q}-\delta_{y,i}\log f_{i}^{new}\left(h\right)\\ +\sum_{q=1}^{Q}\sum_{h\in {\tilde{H}}_{q}}\sum_{i=1}^{K+Q}-\delta_{y,i}\log f_{i}^{new}\left(h\right) \end{array} \end{array}$$

where $\delta_{y,i}$ is an indicator function that equals 1 if i=y and 0 otherwise. To balance old user knowledge retention and new user adaptation, we combine the two losses into a weighted objective:

(11) $$L=\lambda \cdot L_{\mathrm{KD}}+\left(1-\lambda \right)\cdot L_{\mathrm{CE}},$$

where λ controls the trade-off between preserving old knowledge and new users’ adaptation. Although using a fixed λ provides a basic balance, it may limit the model’s long-term flexibility as more new users join. To address this, we propose a dynamic adjustment strategy:

(12) $$\lambda =\min(\lambda_{\max},\lambda_{\mathrm{cur}}+Q\cdot \Delta \lambda ),$$

where $\lambda_{\mathrm{cur}}$ is the current weight, Δλ is a step size, and $\lambda_{\max}$ is an upper bound. As the number of new users increases, λ is also adjusted. After the CIL procedure, the updated model $f^{new}$ is deployed for online authentication.

To provide a clearer overview of the entire procedure, we summarize the PSG-CIL framework in Algorithm 1. This algorithm integrates the three main components described above, including parameter-free pseudo-data generation, confidence-based filtering, and class incremental updating with dynamic weight adjustment.
**Algorithm 1:** PSG-CIL: Parameter-Free Statistical Generator-based Class Incremental Learning
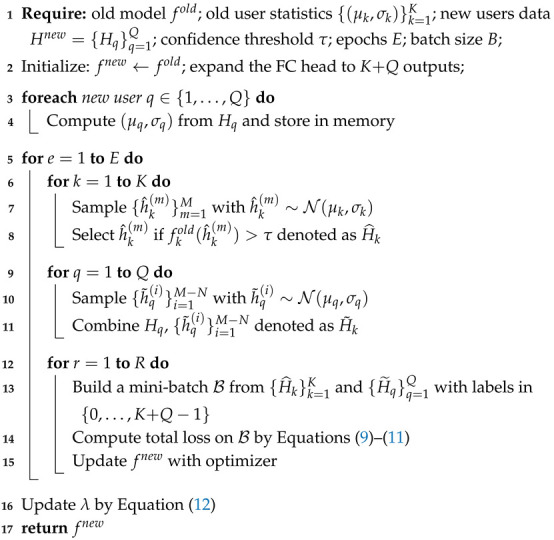


## 5. Performance Evaluation and Simulation Results

In this section, we evaluate the performance of the PSG-CIL framework through extensive experiments.

### 5.1. Real IIoT Scenarios

We conducted our experiments on the AAP open-source dataset provided by the National Institute of Standards and Technology (NIST) [20]. The AAP dataset represents a large industrial factory scenario with dimensions of 400 × 400 × 12 m. CIR data was collected using a fixed transmitter and a mobile receiver that followed predefined trajectories. The transmitter continuously generated a Pseudo-Noise (PN) sequence of 2047 symbols, and the receiver estimated the CIR by correlating the received signal with the PN sequence at a four-times oversampling rate, resulting in each CIR consisting of 8188 complex taps. To evaluate the performance of our method, we also switch the locations of transmitters and receivers according to the channel reciprocity followed by [32,33]. To ensure the generalizability of our results, we selected two representative trajectories from the AAP dataset: the inner loop route and the outer loop route.

#### 5.1.1. Inner Loop Route

**Figure 3:**
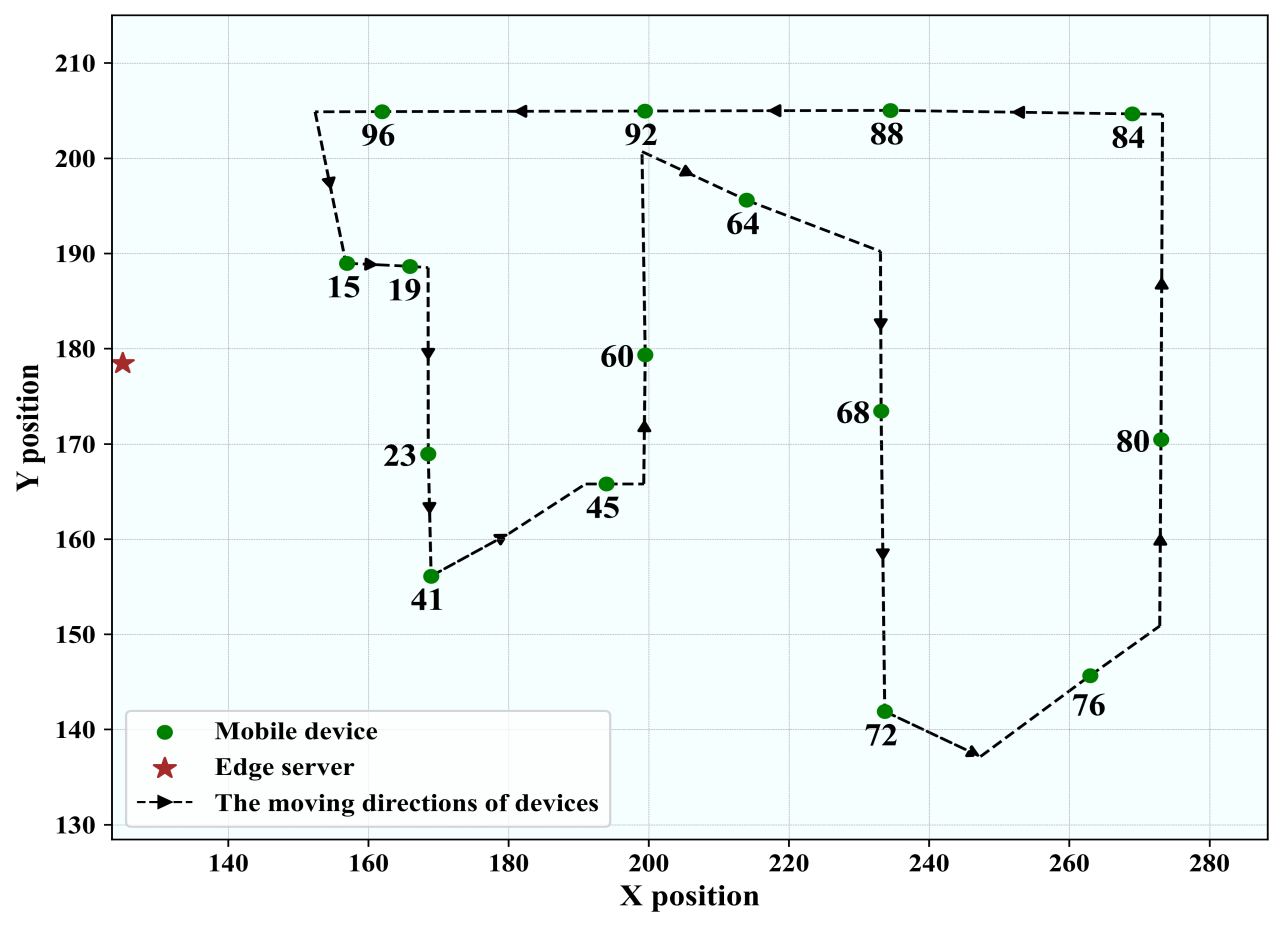
The users’ trajectory map for the inner loop route.

As shown in Figure 3, the inner loop route covers a smaller spatial area compared to the outer loop route, with a maximum distance of approximately 125 m. Most measurement points are within 50 m of the transmitting antenna. The mobile receiver’s trajectory passes through complex industrial environments, including reflective metal surfaces, machine corridors, and track systems. Following [6,27], we selected 15 specific measurement nodes (15, 19, 23, 41, 45, 60, 64, 68, 72, 76, 80, 84, 88, 92, 96) from the AAP dataset to represent different mobile users. The edge server (Bob) is located at coordinates (124.94, 178.46). These nodes were carefully selected to cover diverse propagation conditions in the industrial environment. In particular, they include regions close to reflective metal surfaces, machine corridors, and track systems, where multipath propagation and shadow fading are significant.

#### 5.1.2. Outer Loop Route

**Figure 4:**
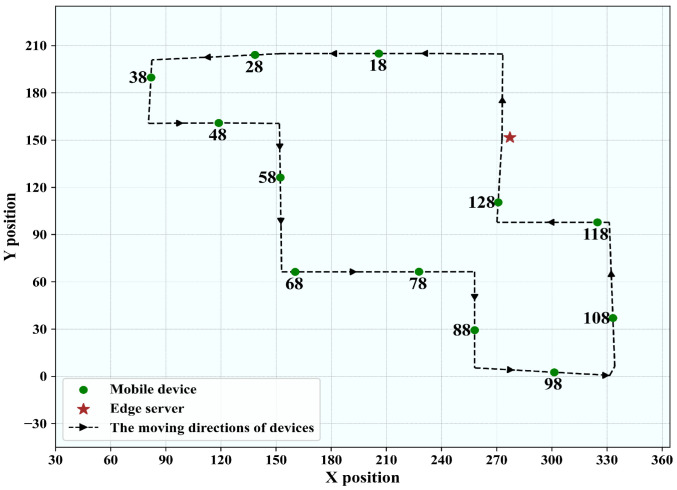
The users’ trajectory map for the outer loop route.

As shown in Figure 4, the outer loop route covers a larger area, with a maximum distance of approximately 200 m. It contains measurement points located at significantly greater distances from the transmitter, where large-scale fading and path loss effects become more pronounced. Due to unpredictable pauses of the mobile receiver during measurements, we selected 12 specific nodes (18, 28, 38, 48, 58, 68, 78, 88, 98, 108, 118, 128) from the AAP dataset to represent different mobile users. The edge server (Bob) is located at coordinates (274.17, 151.64). The selected nodes span varying distances from the transmitter, including both near-field and far-field positions. This allows us to capture different large-scale fading and path loss conditions, which are more pronounced in the outer loop route. By incorporating both closer and farther measurement points, the dataset reflects the broader range of propagation characteristics encountered in real IIoT deployments.

Both datasets contain 300 time slots, yielding 300 CIR records for each user. To simulate real-world applications and ensure the effectiveness of the proposed method, we uniformly utilize N=30 records per user for training, while the remaining samples are reserved for authentication.

### 5.2. Implementation Setting

To evaluate the effectiveness of the proposed PSG-CIL framework compared with other methods, we adopt a standard CNN architecture as the baseline model [6,34], consisting of two convolution layers with a kernel size of 5, max-pooling with stride of 2, and an FC layer that outputs a 256-dimensional feature, providing a strong yet practical benchmark for CIR-based authentication. CNNs are particularly suitable for this task as they can effectively capture multipath structures while maintaining computational efficiency, which is critical for latency-sensitive IIoT scenarios. The parameters were selected to strike a balance between representation capability and model complexity. Nevertheless, as demonstrated in our ablation analysis, PSG-CIL can also be seamlessly integrated with various other deep architectures, such as DNN [5], LPCNN [6], and ResNet [7]. The initial training phase is conducted with K=3 users, using a batch size of B=50 and the Adam optimizer with a learning rate of 0.001. To closely mimic real-world IIoT deployment, we introduce new users incrementally with Q=1 (i.e., one user at a time) rather than adding multiple users simultaneously. This setup better reflects the dynamic nature of IIoT scenarios, where users are not introduced in bulk but gradually integrated into the existing system over time. During the CIL phase, we maintain the Adam optimizer but apply differentiated learning rates: 0.001 for the feature extractor and 0.005 for the FC layer. The batch size remains B=50. To accommodate resource-constrained IIoT settings, we adopt the CIR tap sampling strategy [35] to reduce the CIR length to L=256, while limiting each user’s final dataset size to M=50. The confidence threshold for pseudo-data selection was empirically set to τ=0.8, ensuring a reasonable trade-off between reliability and diversity. As for the dynamic forgetting weight, we initialize λ=0.1, increment it by Δλ=0.025, and cap it at $\lambda_{\max}=0.4$.

### 5.3. Comparison Methods

To evaluate the authentication performance of our proposed PSG-CIL framework, we compare it against five baseline methods: retraining from scratch (RFS), retraining from scratch with parameter-free statistical generator augmentation (RFS++), fine-tuning, Learning Without Forgetting (LWF) [11], and GAN-based. RFS serves as a strong baseline, where the model is entirely retrained from scratch each time a new user is introduced, using the data of both old and new users. RFS++ extends RFS by incorporating our proposed PSG to augment training data for all users, to validate the effectiveness of PSG in mitigating data scarcity. Fine-tuning follows a naïve CIL approach, where the FC layer is expanded for new users, but the model is fine-tuned only with the new user’s data, making it prone to catastrophic forgetting. LWF improves upon fine-tuning by introducing knowledge distillation as a constraint to prevent catastrophic forgetting while adapting to new users. The GAN-based method employs a generative adversarial network to produce pseudo-data for old users, aiming to alleviate forgetting by replaying generated pseudo-data during incremental learning. Specifically, we used a conditional GAN [14] with an MLP-based generator and discriminator. The generator takes a 64-dimensional latent vector concatenated with embedded class labels and maps them through two fully connected layers, while the discriminator processes the concatenation of input data and class labels through two fully connected layers. Training was performed with the Adam optimizer at a learning rate of 0.0002 using binary cross-entropy loss. When new classes arrive, both the generator and discriminator are fine-tuned with the data of the new users, while pseudo-data of the old users are replayed to mitigate forgetting and retain previously learned knowledge.

### 5.4. Evaluation Metrics

To evaluate the performance of the multi-user authentication system, we adopt authentication accuracy (Acc) as the evaluation metric. It is defined as the proportion of correctly classified samples over the total number of samples, expressed as:

(13) $$\mathrm{Acc}=\frac{1}{M}\sum_{m=1}^{M}1\left(p_{m}=y_{m}\right),$$

where *M* denotes the total number of samples, $p_{i}$ is the predicted label of the *i*-th sample, $y_{i}$ is the true label of the *i*-th sample, and 1(·) is the indicator function that returns 1 if $p_{i}=y_{i}$ and 0 otherwise.

### 5.5. Performance Analysis

**Figure 5:**
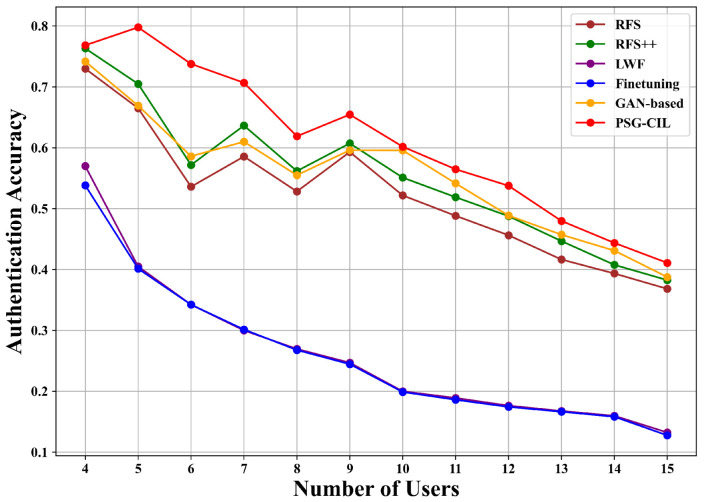
Authentication performance under the inner loop route of the AAP scenario with varying numbers of incremental users.

**Figure 6:**
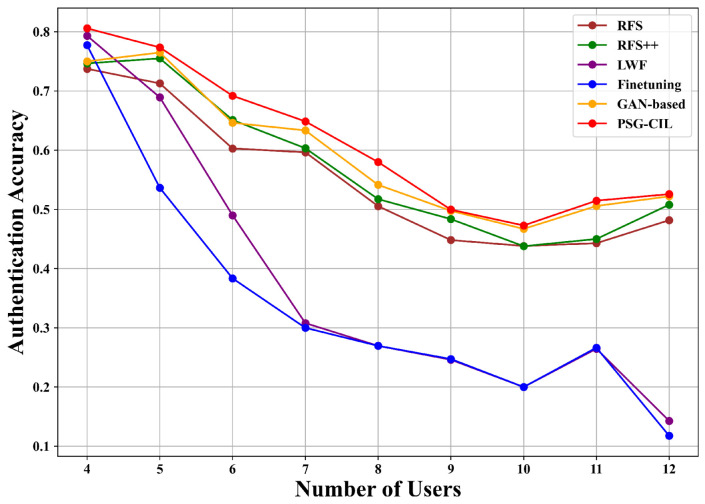
Authentication performance under the outer loop route of the AAP scenario with varying numbers of incremental users.

Figure 5 and Figure 6 present the authentication accuracy of RFS, RFS++, fine-tuning, LWF, GAN-based, and PSG-CIL under outer and inner loop routes, where the number of users corresponds to the total number of authentication classes. In both routes, PSG-CIL consistently achieves the highest accuracy at every stage. For instance, when the number of users reaches seven, RFS and RFS++ achieve accuracies of 58.57% and 63.56% in the outer loop route, whereas PSG-CIL substantially outperforms them with 70.68%. Similarly, in the inner loop route, RFS and RFS++ achieve 59.65% and 60.33%, while PSG-CIL reaches 64.87%, again demonstrating superior performance. The improvement of RFS++ over RFS further confirms that PSG effectively augments user data to enhance model performance. Among the incremental learning baselines, fine-tuning, which updates the model only with new user data, suffers from a sharp decline in accuracy for old users as the total number of users increases, exhibiting severe catastrophic forgetting. LWF partially alleviates this issue by leveraging knowledge distillation to align the new model’s predictions with the old model, but its effectiveness diminishes in later stages due to the limited data for new users. The GAN-based method improves upon LWF by generating pseudo-data for old users, which helps maintain higher accuracy even as the number of users grows, occasionally approaching or surpassing RFS. However, its reliance on parameterized generative models introduces substantial computational overhead, leading to increased training time and model size. As shown in Table 1, GAN-based contains 3.12 M parameters and occupies 11.93 MB storage, which is significantly larger than the other methods (2.18 M parameters and 8.31 MB). This overhead makes GAN-based less suitable for resource-constrained IIoT devices. In contrast, PSG-CIL achieves superior authentication accuracy without increasing model complexity, maintaining the same lightweight scale as conventional methods. This balance between performance and efficiency makes PSG-CIL a practical and robust solution for physical layer authentication in IIoT scenarios.

### 5.6. Ablation Analysis

#### 5.6.1. Comparison Using Different Deep Learning Models

**Figure 7:**
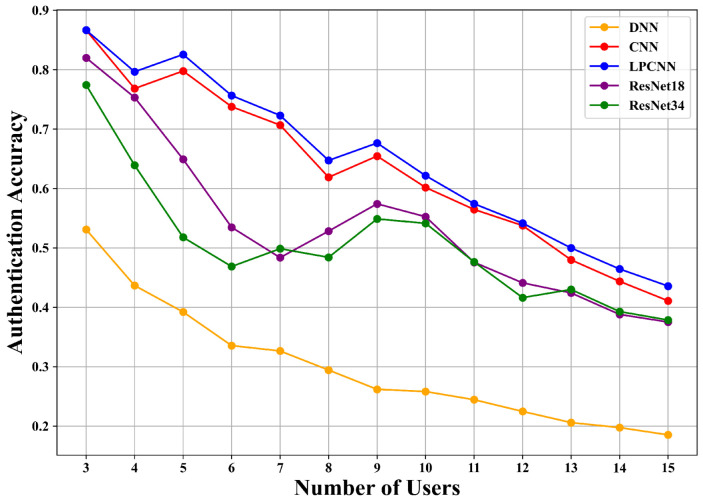
Comparison using different deep learning models on the inner loop route of the AAP scenario.

**Figure 8:**
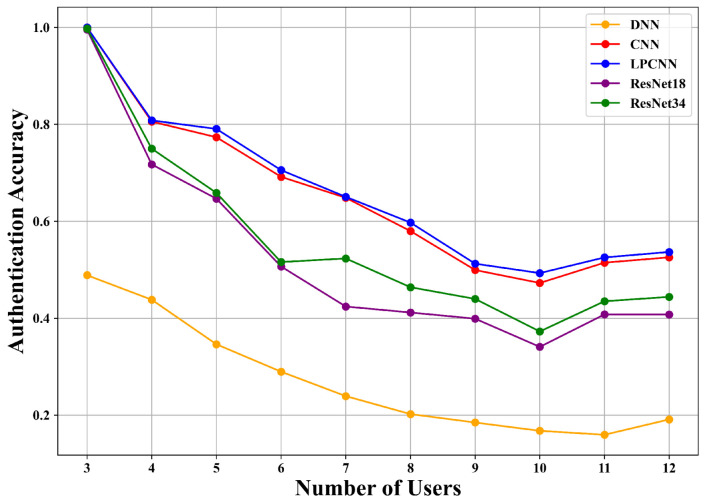
Comparison using different deep learning models on the outer loop route of the AAP scenario.

To further evaluate the adaptability of our PSG-CIL framework, we conducted experiments with multiple backbone architectures, including DNN [5], CNN [34], LPCNN [6], ResNet18 [7], and ResNet34 [7], under consistent experimental settings in both outer and inner loop routes. The results are illustrated in Figure 7 and Figure 8. Among these models, DNN exhibits the weakest performance, while lighter models such as CNN and LPCNN consistently outperform deeper architectures like ResNet18 and ResNet34. This is mainly attributed to two factors: (i) the limited training data available for new users in IIoT scenarios restricts the ability of deeper networks to fully exploit their representational capacity, leading to underfitting; and (ii) the relatively low structural complexity of CIR features reduces the necessity for advanced feature extraction, diminishing the benefits of deeper architectures. These observations highlight that PSG-CIL can be seamlessly integrated with different deep learning models, but CNN and LPCNN demonstrate superior adaptability by achieving higher accuracy with lower complexity. This makes them particularly suitable for incremental multi-user authentication in resource-constrained IIoT scenarios, where both accuracy and efficiency are critical.

#### 5.6.2. Comparison of Dynamic Adjustment and Fixed λ

**Figure 9:**
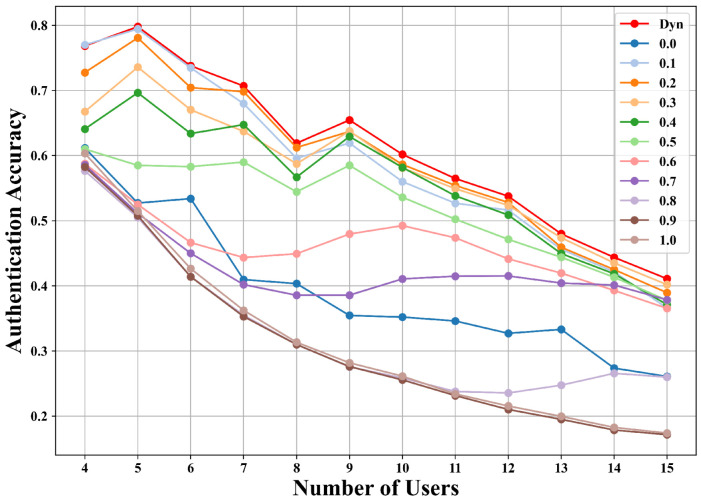
Comparison of dynamic adjustment and fixed λ strategies on the inner loop route of the AAP scenario.

**Figure 10:**
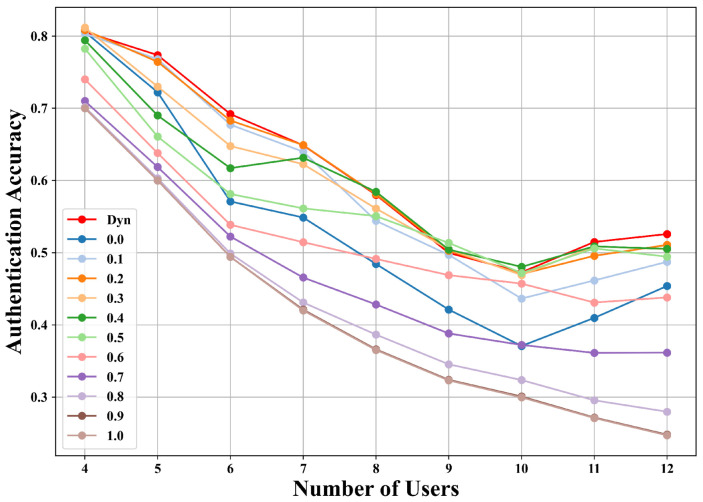
Comparison of dynamic adjustment and fixed λ strategies on the outer loop route of the AAP scenario.

To investigate the effect of the forgetting weight λ, we compare the proposed dynamic adjustment strategy with fixed λ values ranging from 0.0 to 1.0 under both outer and inner loop routes. As shown in Figure 9 and Figure 10, the dynamic adjustment consistently achieves nearly the highest accuracy at all stages and clearly outperforms fixed settings. For example, in the outer loop route with seven users, dynamic adjustment reaches 70.68%, compared to 67.98% with fixed λ=0.1 and 40.20% with fixed λ=0.7. Similarly, in the inner loop route, dynamic adjustment achieves 64.86%, surpassing fixed λ=0.1 at 63.98% and fixed λ=0.7 at 46.55%. Beyond achieving higher authentication accuracy, dynamic adjustment also provides greater stability across incremental stages, avoiding the sharp accuracy drops often observed with fixed λ. Fixed settings generally struggle to balance old and new user knowledge: a smaller λ emphasizes learning about new users but sacrifices retention of old users, whereas a larger λ preserves old knowledge but hinders adaptation to new users. By contrast, dynamic adjustment gradually increases λ as new users are introduced, enabling the model to retain prior knowledge while effectively adapting to an expanding user base.

## 6. Conclusions

In this work, we proposed PSG-CIL, a novel parameter-free CIL framework for multi-user PLA in resource-constrained IIoT scenarios. Unlike conventional generative replay-based approaches that rely on computationally intensive generative models, PSG-CIL employs Gaussian sampling based on user-specific statistical features to generate pseudo-data, thereby eliminating the need for additional parameter training. To enhance the reliability of pseudo-data and improve model adaptability, we introduced a confidence-based selection mechanism and a dynamic loss weight adjustment strategy, which effectively balance the retention of previously learned knowledge and the adaptation to new users. Experimental results on real-world IIoT datasets demonstrate that PSG-CIL consistently achieves higher authentication accuracy compared to existing methods while significantly reducing computational overhead. Although PSG-CIL effectively addresses data scarcity and catastrophic forgetting in IIoT authentication, its benefits may diminish when abundant labeled data are available for each user, or when channel statistics vary too abruptly. In the future, we aim to extend PSG-CIL for more complex channel conditions and to scale it for large-scale user authentication in IIoT scenarios, and investigate advanced pseudo-sample generation mechanisms as well as adaptive or structure-aware modeling strategies to further improve robustness and efficiency.

## Figures and Tables

**Table 1:** Comparison of the parameters and storage size of different methods.

Method	Parameters (M)	Storage Size (MB)
RFS	2.18	8.31
RFS++	2.18	8.31
LWF	2.18	8.31
Fine-tuning	2.18	8.31
GAN-based	3.12	11.93
**PSG-CIL**	2.18	8.31

## Data Availability

The data used to support the findings of this study are available from the corresponding author upon request.

## Abbreviations

The following abbreviations are used in this manuscript:

AAP	Automotive Assembly Plant
CIL	Class Incremental Learning
CIR	Channel Impulse Response
CNN	Convolutional Neural Network
CSI	Channel State Information
DL	Deep Learning
DNN	Deep Neural Network
FC	Fully Connected (layer)
GAN	Generative Adversarial Network
IIoT	Industrial Internet of Things
KD	Knowledge Distillation
LPCNN	Lightweight Perturbed Convolutional Neural Network
LWF	Learning Without Forgetting
PLA	Physical Layer Authentication
PN	Pseudo-Noise
PSG	Parameter-Free Statistical Generator
PSG-CIL	Parameter-Free Statistical Generator-based Class Incremental Learning Framework
RFS	Retraining From Scratch
RFS++	Retraining From Scratch with Parameter-Free Statistical Generator Augmentation
ResNet	Residual Network
VAE	Variational Autoencoder
